# Green Synthesis of Privileged Benzimidazole Scaffolds Using Active Deep Eutectic Solvent

**DOI:** 10.3390/molecules24162885

**Published:** 2019-08-08

**Authors:** Maria Luisa Di Gioia, Roberta Cassano, Paola Costanzo, Natividad Herrera Cano, Loredana Maiuolo, Monica Nardi, Fiore Pasquale Nicoletta, Manuela Oliverio, Antonio Procopio

**Affiliations:** 1 Dipartimento di Farmacia e Scienze della Salute e della Nutrizione, Dipartimento di Eccellenza L. 232/2016, Edificio Polifunzionale, Università della Calabria, 87036 Rende (CS), Italy; 2 Department of Health Sciences, Magna Græcia University, Viale Europa, 88100 Germaneto CZ, Italy; 3 ICYTAC, CONICET and Universidad Nacional de Córdoba, Facultad de Ciencias Químicas, Departamento. Química Orgánica. Ciudad Universitaria, Bv. Juan Filloy s/n, 5000 Córdoba, Argentina; 4 Dipartimento di Chimica e Tecnologie Chimiche, Via P. Bucci, cubo 12C, Università della Calabria, 87036 Rende (CS), Italy

**Keywords:** benzimidazoles, deep eutectic solvents, green chemistry, aromatic amines, heterocyclic moiety

## Abstract

The exploitation and use of alternative synthetic methods, in the face of classical procedures that do not conform to the ethics of green chemistry, represent an ever-present problem in the pharmaceutical industry. The procedures for the synthesis of benzimidazoles have become a focus in synthetic organic chemistry, as they are building blocks of strong interest for the development of compounds with pharmacological activity. Various benzimidazole derivatives exhibit important activities such as antimicrobial, antiviral, anti-inflammatory, and analgesic activities, and some of the already synthesized compounds have found very strong applications in medicine praxis. Here we report a selective and sustainable method for the synthesis of 1,2-disubstituted or 2-substituted benzimidazoles, starting from *o*-phenylenediamine in the presence of different aldehydes. The use of deep eutectic solvent (DES), both as reaction medium and reagent without any external solvent, provides advantages in terms of yields as well as in the work up procedure of the reaction.

## 1. Introduction

**Figure 1:**
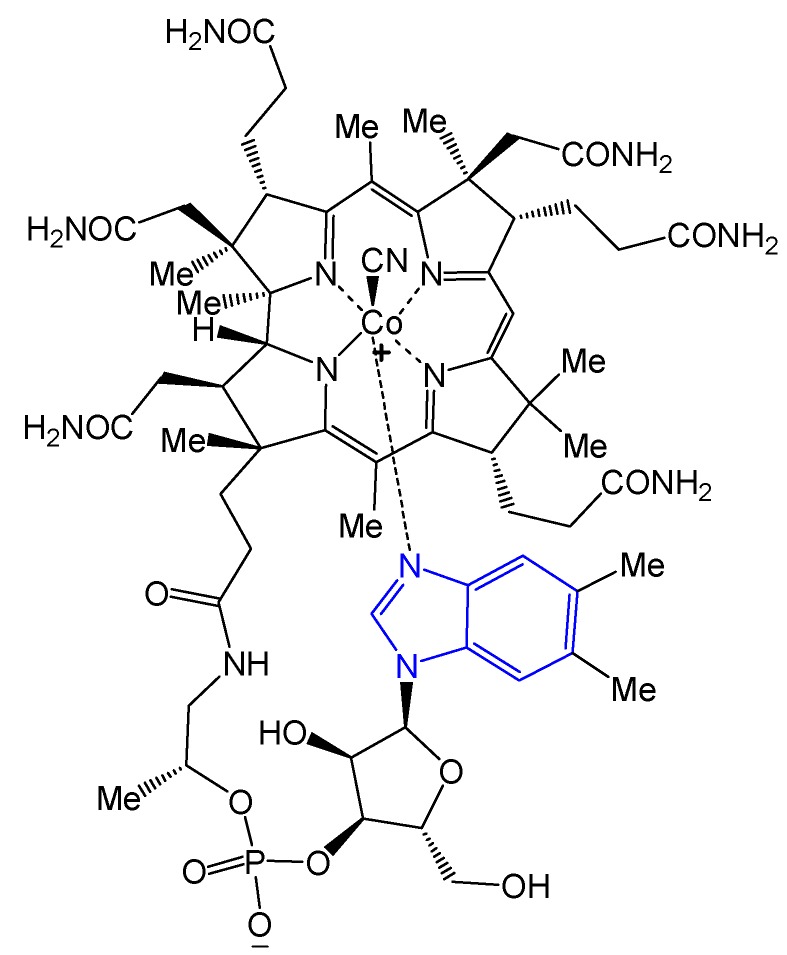
Benzimidazole nucleus in vitamin B_12_.

Among the heterocyclic pharmacophores, the benzimidazole ring is one of the most widespread systems in nature. It is indicated as a “privileged nucleus” due to its occurrence in molecules essential for the life cycle of organisms [1]. The 5,6-dimethylbenzimidazole moiety in the structure of vitamin B_12_ [2] is an important example (Figure 1).

**Figure 2:**
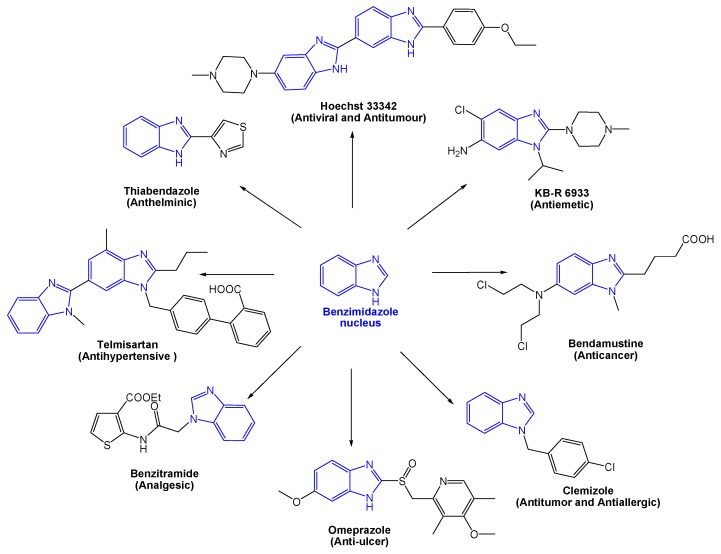
Examples of important drugs containing a benzimidazole nucleus.

Bioactive compounds with a benzimidazole nucleus are heterogeneous molecules in structure and activity. This diversification is to be found in the derivatization of the basic core, followed by a structure–activity relationship for each compound. The first example of a clinically available benzimidazole-based drug is thiabendazole, capable of acting as a fungicide and antiparasitic [3]. Over the years, many other derivatives have been developed: The antihistamine Clemizole, the anti-ulcerative Omeprazole, the antihypertensive Telmisartan, antifungal Thiabendazol, analgesic Bezitramide, antiviral Hoechst 33342, anticancer Bendastumide, and antiemetic KB-R-6933 (Figure 2) [4].

More recently, the treatment potency of benzimidazoles in diseases such as ischemia-reperfusion injury or hypertension, have also been reported [5].

Due to their properties and roles in various diseases, special interest has been devoted to benzimidazole-based chemistry [6,7,8,9]. A lot of synthetic methodologies are available for the preparation of benzimidazole and its derivatives. Generally, the reaction between *o*-phenylenediamines and carboxylic acids or their derivatives has been used [10,11].

A different and widely used procedure for the same synthesis is the condensation of *o*-phenylenediamine with differently substituted aldehydes affording 2-substituted and 1,2-di-substituted benzimidazoles derivatives. However, these protocols suffer some drawbacks such as long reaction times, expensive reagents, use of toxic organic solvents, difficulties in the preparation of the catalyst, non-recoverability of the catalyst, and tedious work-up procedures. Moreover, most of them lack selectivity [12,13,14,15,16,17]. Therefore, the introduction of simple, efficient, and mild procedures with easily separable and recyclable catalysts, and in particular, greater selectivity is still in demand. Recently, the use of water [18,19,20,21] or ionic liquids (ILs) as green media, and/or the use of readily available organometallic catalysts, have been exploited [22,23,24,25,26,27,28].

Although these protocols provide improvement, it is well-known that ILs are (eco)toxic and harmful to the environment [29]. Further, their synthesis and purification is often expensive and time-consuming [30].

In the last decade, the most important drug manufacturing industries have been influenced by green chemistry principles introducing “greener” raw materials, less use of toxic organic solvents, cuts in waste production, and alternative organic synthetic methods [31].

In this regard, as the pharmaceutical industry is known to use a large amount of solvents to produce active pharmaceutical ingredient (API), most of the investigations are currently focusing on the replacement of hazardous conventional solvents with more sustainable alternatives such as water [32,33,34,35,36,37,38,39,40], supercritical fluids [41,42], ionic liquids [43,44,45,46,47,48,49,50], and solvents derived from biomass [51,52,53].

Deep eutectic solvents (DES) are considered the green solvents of the 21st century with tremendous applicability in all areas of the chemical industry [54]. They can be defined as a mixture of two or more compounds, that at certain molar ratios exhibit a high depression of the melting point, becoming liquid at or near room temperature. At these conditions, the compounds that form the deep eutectic solvents interact between themselves, mainly through hydrogen bonding, thus enabling the components to behave as one single entity [55,56,57].

Because the production of these deep eutectic solvents relies solely on the physical mixture of two or more natural components, their production has virtually no impact on the environment. Moreover, because deep eutectic solvents do not need any complex processing and equipment, they are also cheap alternatives to most common green solvents such as ionic liquids [55].

In our continuous efforts towards green organic chemistry, here we present a new synthetic route to benzimidazole derivatives. The novel feature of the procedure proposed is that in the first step, a DES is formed consisting of *o*-phenylendiamine (*o*-PDA) and choline chloride (ChCl). Therefore, we explored a double role of the DES: Solvent and reactant.

## 2. Results and Discussion

The pilot reaction between *o*-phenylenediamine and benzaldehyde, as reported in literature, involves indiscriminately the formation of monosubstituted and disubstituted benzimidazole derivatives: It is a nonselective synthesis [58].

Thus, we started our investigation conducting the model reaction with the most explored DES choline chloride/urea (ChCl:urea) as an eco-alternative solvent.

Choline chloride is one of the most commonly used hydrogen bond acceptors (HBA) used for the formation of DES [56] and its combination with a suitable HBD (usually sugars, natural organic acids, amides, etc.) produces eutectic mixtures that are liquid at ambient temperature and have unusual solvent properties [57].

It is an economic, biodegradable, nontoxic, and even edible quaternary salt that can be extracted from biomass or easily synthesized from fossil reserves. Similarly, urea has a low cost, is easily available, and is absolutely nontoxic. Both compounds are constitutively present in our body. Choline is a ubiquitous molecule, mainly responsible for the structural integrity of cell membranes (it is a component of membrane phospholipids) and for the synthesis of the neuronal chemical mediator acetylcholine; urea is one of the products of protein metabolism, mainly developed by the liver and kidneys, and eliminated by them. A solvent characterized by such a composition can only be defined as absolutely nontoxic. It is noteworthy that to obtain the eutectic mixture, the two compounds are simply mixed and left in the right heating conditions; urea, through the formation of hydrogen bonds, is easily associated with choline chloride.

The model reaction was performed dissolving *o*-phenylenediamine (1 mmol) in 1.0 mL of ChCl:urea DES and then adding benzaldehyde in an equimolar ratio. The reaction mixture was left under magnetic stirring at 60 °C or 80 °C and monitored by thin layer chromatography (TLC) and gas chromatography/mass spectrometry (GC/MS) analysis. The GC/MS analysis confirmed the complete conversion of the reagents within 10 min affording, at the higher temperature, the 2-substituted benzimidazole derivative **1a** and the 1,2-disubstituted benzimidazole derivative **1b** in 88% and 12% yields respectively (entries 1 and 2 in Table 1).

The reaction in DES was replicated by varying the molar ratios of the reagents using the diamine and benzaldehyde in a 1:2 ratio. In this case, a selectivity towards the double-condensation product **b** was observed by GC/MS analysis. After 10 min of reaction the conversion of the reagents went almost completely in favor of the product **b** that was obtained in 87% yield while **a** was afforded in 13% yield.

The same reaction performed in DES at 60 °C provided, after 15 min, a mixture of products **a** and **b** both in the case of a molar ratio diamine:benzaldehyde 1:1 and in the case of the molar ratio 1:2 (Table 1, entries 1 and 2).

The high selectivity observed in DES let us hope that we had developed one of the most promising and green methods for obtaining benzimidazole derivatives. DESs as an alternative to ILs, not only have similar characteristics to traditional imidazolium based ILs, but also have several benefits such as ease of preparation and availability from bulk renewable resources. In addition, the use of choline chloride/urea enables an easy work-up as the products can be recovered by simple extraction with ethyl acetate, followed by separation and removal of the solvent under reduced pressure.

To further improve our green procedure, we decided at this stage to explore a double role of the DES. In fact, the use of solvents as reagents is an efficient and widely explored way to minimize waste formation [59]. The use of urea to form type III eutectic solvents where choline chloride is mostly taken as quaternary ammonium cation has been known for a considerable time. This principle, however, is not limited to amides, but can be applied to a wide variety of other hydrogen bond donors (HBDs) such as organic acids, alcohols, and amines. To date, only a few example of DESs based on ChCl and amine group bearing compounds have been reported [60,61] and only one example of DESs based on ChCl and solid aromatic amines has been described [62]. Nevertheless, none of these DESs have been used for organic synthetic applications.

In the light of the above statement, our approach was to explore *o*-phenylendiamine (*o*-PDA) as HBD to combine with choline chloride, thus forming a eutectic mixture: The diamine would therefore be part of the solvent and at the same time reactant.

**Figure 3:**
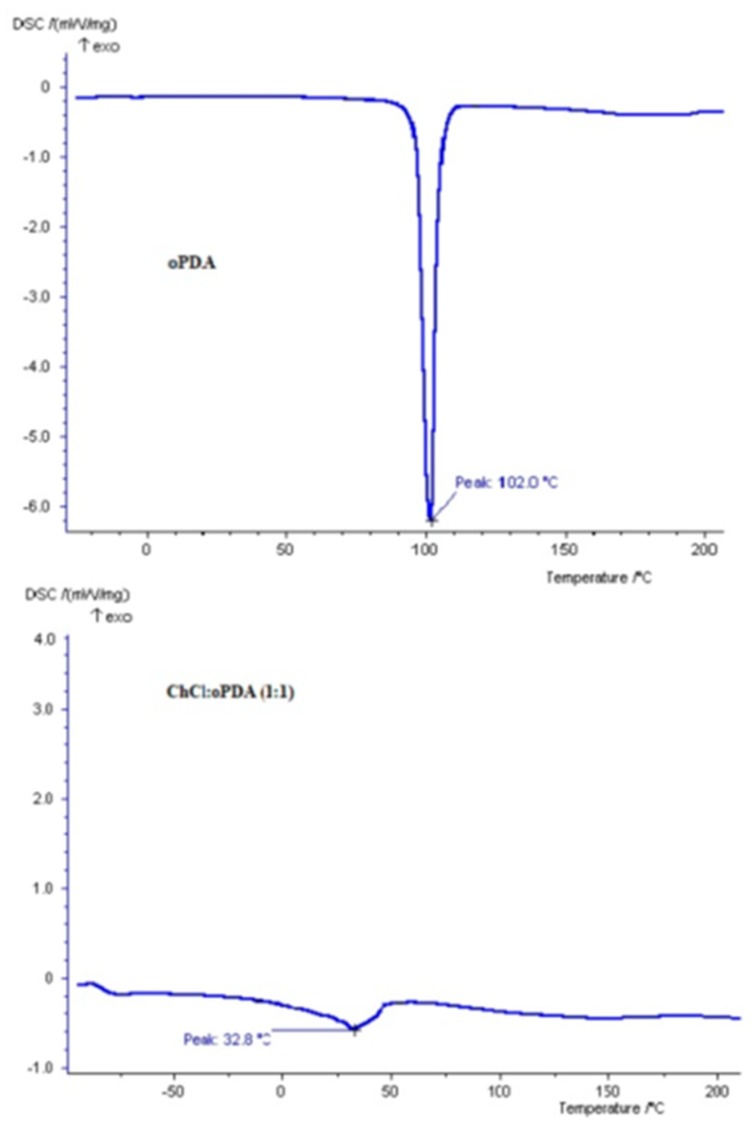
Eutectic temperature (T_f_) of ChCl with *o*-phenylendiamine (*o*-PDA). The melting point of pure ChCl is 302 °C.

Thus, a ChCl: *o*-PDA based DES was prepared by mixing the components in a molar ratio amine: ChCl 1:1 at ambient temperature and then heated up to 80 °C for 2 h, to obtain a liquid product. The final mixture was a light yellow colored liquid, gradually becoming green on air, and that after preparation slightly increased its viscosity. A differential scanning calorimetry (DSC) analysis of the mixture was performed, as well as for the individual components, to demonstrate the formation of the DES. ChCl and *o*-phenylendiamine are solid components melting at 302 °C and 102 °C, respectively: The DSC analysis of the mixture resulted in a eutectic that melts at 32 °C. This result demonstrated the successful formation of a eutectic with a melting point significantly lower than that of its individual components. The eutectic temperature for ChCl: *o*-PDA DES is shown in Table 2 and graphically in Figure 3.

In the thermogram of *o*-phenylendiamine melting is observed with T_m_ of 102 °C. From the thermogram of ChCl:*o*–PDA (1:1) DES shown in Figure 3 it is clear that the thermal behavior of the DES is different from the behavior of the individual components, thus providing evidence of interaction established between choline chloride and the diamine, which make the DES behave like a different entity.

Abbott et al. suggested that the depression of the freezing point is dependent on the lattice energies of the DESs and low melting mixtures (LMMs), the interaction of the anion and HBD, and the entropy changes arising from forming a liquid [63,64,65]. In our situation, the influence of the amine structure on the physical properties of ChCl/*o*-PDA can be interpreted based on available results for DESs formed by ChCl mixtures with other aromatic hydrogen bond donors, such as phenols [62,66]. In fact, Zhu et al. [66] showed that the structure of phenols is responsible for hydrogen bond formation between the phenolic group and Cl anion of ChCl. In accordance, Spychaj et al. [62] assumed that aromatic NH_2_ can interact with choline chloride, thus affecting the physicochemical properties of DES.

The obtained DES (ChCl:*o*–PDA) was studied as solvent and, at the same time, reactant in the pilot reaction for the synthesis of benzimidazole derivatives. To this end, 1 mol of benzaldehyde with respect to the DES component *o*-PDA, was added and magnetically stirred for 10 min at 80 °C.

**Scheme 1:**
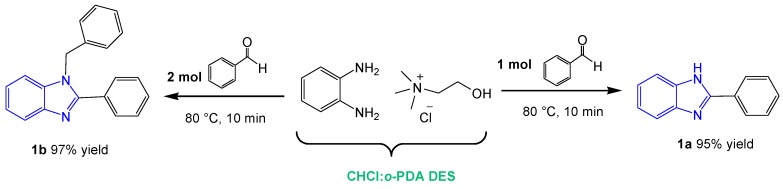
Optimized conditions for the pilot reaction. GC/MS analysis showed the formation of compound **a** as the only product (95% yield) when using 1 mol of benzaldehyde and the formation of compound **b** as the only product (97% yield) when using 2 mol benzaldehyde.

GC/MS analysis of the mixture revealed the formation of compound **1a** as the only product of reaction (95% yield, Scheme 1). Employing 2 mol of benzaldehyde for the same reaction, the 1,2-disubstituted benzimidazole **1b** was selectively obtained in 97% yield as a single product (Scheme 1). The reaction conditions were finally optimized as follows: 1 mol benzaldehyde in ChCl:*o*–PDA (1:1) DES at 80 °C to give the monosubstituted benzimidazole derivative **1a** (Scheme 1); 2 mol benzaldehyde in the same reaction conditions to afford the 1,2-disubstituted benzimidazole **1b** (Scheme 1).

An important advantage of this solvent system is that the use of the new DES, enables an easy work-up without using any chromatographic or other purification methods. In fact, the reaction products were recovered by simple dilution of the mixture with water followed by extraction with ethyl acetate.

The application scope of this reaction was then examined by subjecting different aldehydes to the same protocol. By using this reactive DES solvent and varying the molar ratio of the aldehyde, 2-substituted or 1,2-disubstituted benzimidazoles with various functional groups can be obtained in excellent selectivity and yields (Table 3 and Table 4).

All reactions were complete and the reaction times were always short, generally between 8 and 10 min. The reaction yields related to the formation of the 2-substituted benzimidazoles derivative ranged from 89% to 97%. The reaction yields related to the formation of the disubstituted benzimidazoles derivative were between 91% and 98%.

As it can be seen from Table 3, good reaction yields were obtained with aldehydes containing electron donor groups (entries 2–6), and also electron withdrawing groups (entries 7–8).

The generality of the selective formation of 1,2-disubstituted benzimidazoles was demonstrated through the reaction of *o*-phenylendiamine with 2 molar amount of various aldehydes (Table 4). The expected 1,2-disubstituted products were obtained in 90%–98% yields. The reaction, as already reported in the literature, proceeds through a bis-imine formation, a rearrangement, and a 1,3-hydride shift pathway that finally afford the 1,2-disubstituted derivatives [67]. The DES formed by choline chloride and the diamine possibly increases the electrophilic character at aldehyde carbon, which will facilitate the nucleophilic addition of *o*-phenylenediamine to obtain the benzimidazole derivatives. The intermediate formed could be stabilized through hydrogen bonding and electronic interaction with DES components, thereby exalting the electrophilic character of the aldehydes.

However, the reactions performed with 2 molar amount of aldehydes containing electron withdrawing groups such as *p*-chloro or *p*-nitro benzaldehyde (Table 4, entries 7–8) afforded exclusively the corresponding 2-monosubstituted benzimidazoles (**7a** and **8a**) in good yields, without observing the formation of disubstituted derivative. This result is in accordance with the data reported in the literature [21]. Electron deficient aldehydes, as they possess a lower density of negative charge on the oxygen atom of the carbonyl group, do not simply coordinate with the DES components to form the hydrogen bonding interaction. As a consequence, the formation of the disubstituted product is not favored. Additionally, the highly electrophilic character of the carbonyl carbon of aldehydes with a chloro or nitro substituent is responsible, after the formation of the mono-imine, for the fast formation of the monosubstituted benzimidazole derivative **7a** and **8a** via the corresponding intermediate imidazoline [67].

Finally, in order to demonstrate the potential industrial applicability of this green procedure, the pilot reaction to give **1a** was carried out in a scale of 20 mol (entry 1, Table 3, footnote c). The reaction was completed in 30 min with 93% isolated yield after simple water addition (10 mL) and extraction with 10 mL ethyl acetate.

In the development of a green procedure, solvent recyclability and reusability is an essential feature. In this case, after completion of the reactions, ChCl is dissolved in water and can be recycled easily by water distillation under vacuum. However, as water distillation consumes a lot of energy and is not an advantageous process from an economical point of view and, since choline chloride is a very cheap and nondangerous substance (500 g € 49.80 MERCK, 2019), at the end of reaction the aqueous solution can be simply thrown away.

## 3. Materials and Methods

### 3.1. General Information

All chemicals and solvents were purchased from common commercial sources and were used as received without any further purification. All reactions were monitored by GC/MS analysis and TLC on silica Merck 60 F_254_ precoated aluminum plates (KGaA, Darmstadt, Germany). The GC-MS Shimadzu workstation was constituted by a GC 2010 (equipped with a 30 m-QUADREX 007-5MS capillary column (BGB Analytik AG, CH-4461 Boeckten, Switzerland) operating in “split” mode, 1 mL min^−1^ flow of He as carrier gas) and a 2010 quadrupole mass-detector. Proton nuclear magnetic resonance (^1^H NMR) spectra were recorded on a Brüker spectrometer (Auckland, New Zealand) at 300 MHz. Chemical shifts are reported in δ units (ppm) with TMS as reference (δ 0.00). All coupling constants (J) are reported in Hertz. Multiplicity is indicated by one or more of the following: s (singlet), d (doublet), t (triplet), q (quartet), m (multiplet). Carbon nuclear magnetic resonance (^13^C NMR) spectra were recorded on a Brüker at 75 MHz. Chemical shifts are reported in δ units (ppm) relative to CDCl_3_ (δ 77.0) (more information see Supplementary Materials).

### 3.2. General Procedure for DESs Preparation

The ChCl:urea (1:2) DES was prepared as follows: Choline chloride (6.98 g, 50 mmol) and urea (6.00 g, 100 mmol) were added in a round-bottom flask under inert atmosphere. The mixture was magnetically stirred for 60 min at 80 °C until a clear colorless liquid was obtained. The obtained DES was used without need of purification.

For the preparation of ChCl:*o*–PDA (1:1) DES the following procedure was used: Choline chloride (6.98 g, 50 mmol) and *o*-phenylendiamine (5.40 g, 50 mmol) were mixed in a round-bottom flask under inert atmosphere. The mixture was magnetically stirred for 2 h at 80 °C until a clear yellow liquid was obtained. The obtained DES was characterized by DSC analysis and used without further purification.

### 3.3. General Procedure for the Synthesis of 2-Substituted Benzimidazoles **1a**–**8a** in the DES ChCl:o–PDA (1:1)

The appropriate aldehyde (1 mmol) was added to the ChCl:*o*–PDA (1:1) eutectic mixture (1 mL) under magnetic stirring. The resulting mixture was stirred at 80 °C for 8–10 min. The reaction was monitored by TLC and GC/MS analysis. After this time, 2 mL of H_2_O were added. The resulting aqueous suspension was then extracted with AcOEt (3 × 2 mL). The organic phases were dried over Na_2_SO_4_, followed by evaporation under reduced pressure to give the corresponding products **1a**–**8a**. Spectral data were in accordance with the literature [21].

### 3.4. General Procedure for the Synthesis of 1,2-Substituted Benzimidazoles **1b**–**8b** in the DES ChCl:o–PDA (1:1)

The appropriate aldehyde (2 mmol) was added to the ChCl:*o*–PDA (1:1) eutectic mixture (1 mL) under magnetic stirring. The resulting mixture was stirred at 80 °C for 8–10 min. The reaction was monitored by TLC and GC/MS analysis. After this time, 2 mL of H_2_O were added. The resulting aqueous suspension was then extracted with AcOEt (3 × 2 mL). The organic phases were dried over Na_2_SO_4_, followed by evaporation under reduced pressure to give the corresponding products **1b**–**8b**. Spectral data were in accordance with the literature [21].

### 3.5. Differential Scanning Analysis (DSC)

The ChCl:*o*–PDA DES mixture and raw chemicals were characterized by DSC analysis (model DSC NETZSCH 200) on the temperature range from −80 °C to 350 °C, at 10 °C/min, after equilibration for 5 min at −80 °C. The experiments were performed under nitrogen atmosphere (50 mL/min), with 15 mg of the sample in aluminum pans with covering lids.

## 4. Conclusions

For the first time a type III DES based on ChCl as quaternary ammonium salt and a HBD such as *o*-phenylendiamine (ChCl:*o*–PDA, 1:1) was prepared and used as medium, and at the same time as a reagent, for the synthesis of benzimidazole derivatives.

The methodology proved to be complete in terms of eco-sustainability, ecotoxicity, reaction, and economics.

In summary, it can be asserted that the high reaction yield, the selectivity of the process, the easy preparation of the solvent, the economy, the short reaction times, and the absence of chromatographic purification are the salient aspects of our approach.

The use of easy-to-handle and environment-friendly chemicals with low toxicity and without using any external solvent makes this method a potential approach for obtaining benzimidazole derivatives, even on a large scale, and perfectly fulfills several requirements, as formulated by Anastas et al. in the twelve principles of green chemistry [68].

## Figures and Tables

**Table 1:**
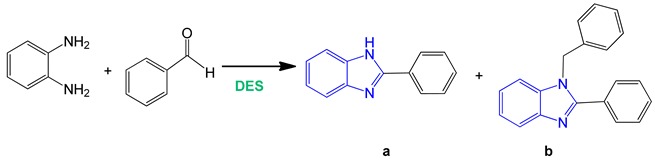
Optimization of the reaction conditions in pilot reaction.

Entry	Solvent	Molar Ratio *o*-PDA:Benzaldehyde	T (°C)	Time (min)	Yield (%) ^3^ a:b
1 ^1^	ChCl:urea (1:2)	1:1	60	15	67:33
2 ^2^	ChCl:urea (1:2)	1:2	60	15	30:70
3 ^1^	ChCl:urea (1:2)	1:1	80	10	88:12
4 ^2^	ChCl:urea (1:2)	1:2	80	10	13:87

^1^ General reaction conditions: *o*-phenylendiamine (1 mmol) and benzaldehyde (1 mmol) were dissolved in 1.0 mL DES and stirred for 10–15 min at different temperatures. ^2^ General reaction conditions: *o*-phenylendiamine (1 mmol) and benzaldehyde (2 mmol) were dissolved in 1.0 mL DES and stirred for 10–15 min at different temperatures. ^3^ The complete conversion of the reagents was observed. Ratio (a:b) determined by GC/MS.

**Table 2:** Structure, composition, and eutectic temperature (T_f_) of the deep eutectic solvents (DES) with the corresponding melting point (T_m_*) of the pure hydrogen bond donor (HBD). The temperature is given in °C.

ChCl	HBD	Molar Ratio	T_f_ (°C)	T_m_ HBD (°C)	Δ (°C)	Appearance
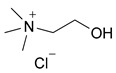	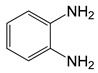	1:1	32	102	70	Light yellow liquid that tends to become greenish.

**Table 3:**
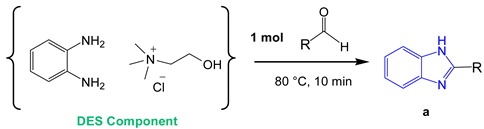
Synthesis of 2-substituted benzimidazoles ^a^.

Entry	R	Product		Yields ^b^ (%)
1	Ph	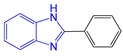	**1a**	95 (93) ^c^
2	*p*-MePh	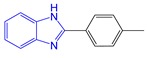	**2a**	97
3	*p*-MeOPh	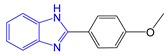	**3a**	92
4	CH_3_CH_2_	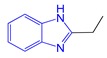	**4a**	95
5	CH_3_	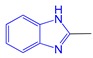	**5a**	96
6	CH_2_Ph	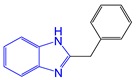	**6a**	91
7	*p*-ClPh	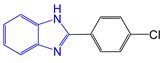	**7a**	90
8	*p*-NO_2_Ph	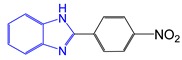	**8a**	89

^a^ General reaction conditions: 1 mmol of aldehyde was dissolved under stirring in ChCl: *o*-PDA (1:1) DES at 80 °C for 10 min. ^b^ Percent yield calculated from GC/MS data. The corresponding monosubstituted benzimidazole derivative was recovered as the sole product. ^c^ The reaction was carried out in a scale of 20 mol and furnished **1a** in 93% yield.

**Table 4:**
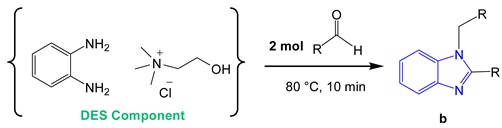
Synthesis of 1,2-disubstituted benzimidazoles ^a^.

Entry	R	Product		Yields ^b^ (%)
1	Ph	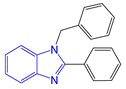	**1b**	97
2	*p*-MePh	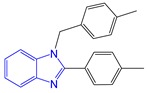	**2b**	98
3	*p*-MeOPh	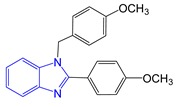	**3b**	93
4	CH_3_CH_2_	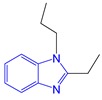	**4b**	90
5	CH_3_	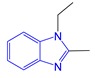	**5b**	91
6	CH_2_Ph	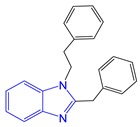	**6b**	91
7 ^c^	*p*-ClPh	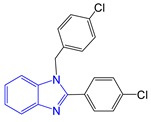	**7b**	0
8 ^c^	*p*-NO_2_Ph	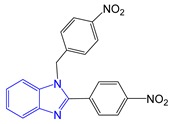	**8b**	0

^a^ General reaction conditions: 2 mol of aldehyde was dissolved under stirring in ChCl:o–PDA (1:1) DES at 80 °C for 10 min. ^b^ Percent yield calculated from GC/MS data. The corresponding disubstituted benzimidazole derivative was recovered as the sole product. ^c^ Product b was not detected. Only the corresponding 2-substituted derivative (**7a** 93% yield and **8a** 93% yield) was afforded.

## Supplementary Materials

The following are available online, MS(EI) spectra of products, DSC thermograms and general synthetic procedures.
