# Supplementary material for: Green Synthesis of Privileged Benzimidazole Scaffolds Using Active Deep Eutectic Solvent
Source: Molecules. 2019 Aug 8;24(16):2885. doi: 10.3390/molecules24162885 (PMC6719900; doi:10.3390/molecules24162885)
Supplement: Supplementary file 1 [file molecules-24-02885-s001.pdf]

Green Synthesis of Privileged Benzimidazole Scaffolds using Active Deep Eutectic Solvent

Maria Luisa Di Gioia[1,*], Roberta Cassano[1], Paola Costanzo[2*], Natividad Herrera Cano[3], Loredana Maiuolo[4], Monica Nardi [2], Fiore Pasquale Nicoletta,[1] Manuela Oliverio [2] and Antonio Procopio [2]

1 Dipartimento di Farmacia e Scienze della Salute e della Nutrizione, Dipartimento di Eccellenza L. 232/2016, Edificio Polifunzionale, Università della Calabria, 87036 Rende (CS), Italy; ml.digioia@unical.it (M.L.D.G.), roberta.cassano@unical.it (R.C.), nicolett@unical.it (F.P.N.)

2 Department of Health Sciences, Magna Græcia University, Viale Europa, 88100 Germaneto CZ, Italy; pcostanzo@unicz.it (P.C.); monica.nardi@unicz.it (M.N.); m.oliverio@unicz.it (M.O.); procopio@unicz.it (A.P.)

3 ICYTAC, CONICET and Universidad Nacional de Córdoba, Facultad de Ciencias Químicas, Departamento. Química Orgánica. Ciudad Universitaria, Bv. Juan Filloy s/n, 5000, Córdoba, Argentina; nhc@fcq.unc.edu.ar (N.H.C.)

4 Dipartimento di Chimica e Tecnologie Chimiche, Via P. Bucci, cubo 12C, Università della Calabria, 87036 Rende (CS), Italy; maiuolo@unical.it (L.M.);

* Author to whom correspondence should be addressed: ml.digioia@unical.it; Tel.: + 39-0984 493095; pcostanzo@unicz.it; Tel.: +39 0961 3694120

Electronic Supplementary Material

Table of Contents

**Experimental Section**
General Procedure for DESs Preparation
General Procedure for the Synthesis of 2-Substituted Benzimidazoles 1a–8a in the DES ChCl: o-PDA **(1:1).**
General Procedure for the Synthesis of 1,2-Substituted Benzimidazoles 1b–8b in the DES ChCl: o-PDA ***(1:1).***
MS(EI) spectra
Differential scanning analysis (DSC)

Experimental Section

All chemicals and solvents were purchased from common commercial sources and were used as received without any further purification. All reactions were monitored by GC/MS analysis and TLC on silica Merck 60 F_{254} precoated aluminum plates. The GC-MS Shimadzu workstation was constituted by a GC 2010 (equipped with a 30 m-QUADREX 007-5MS capillary column, operating in "split" mode, 1 mL min-1 flow of He as carrier gas) and a 2010 quadrupole mass-detector. Proton nuclear magnetic resonance (^{1}H NMR) spectra were recorded on a Brüker spectrometer at 300 MHz. Chemical shifts are reported in δ units (ppm) with TMS as reference (δ 0.00). All coupling constants (J) are reported in Hertz. Multiplicity is indicated by one or more of the following: s (singlet), d (doublet), t (triplet), q (quartet), m (multiplet). Carbon nuclear magnetic resonance (^{13}C NMR) spectra

were recorded on a Brüker at 75 MHz. Chemical shifts are reported in δ units (ppm) relative to $CDCl_3$ (δ 77.0).

General Procedure for DESs Preparation.

The ChCl:urea (1:2) DES was prepared as follows: Choline chloride (6.98 g, 50 mmol) and urea (6.00 g, 100 mmol) were added in a round-bottom flask under inert atmosphere. The mixture was magnetically stirred for 60 min at 80 °C until a clear colourless liquid was obtained. The obtained DES was used without need of purification.

For the preparation of ChCl:o-PDA (1:1) DES the following procedure was used: Choline chloride (6.98 g, 50 mmol) and o-phenylendiamine (5.40 g, 50 mmol) were mixed in a round-bottom flask under inert atmosphere. The mixture was magnetically stirred for 2 h at 80 °C until a clear yellow liquid was obtained. The obtained DES was characterized by DSC analysis and used without further purification.

General Procedure for the Synthesis of 2-Substituted Benzimidazoles 1a–8a in the DES ChCl:o-PDA (1:1).

The appropriate aldehyde (1 mmol) was added to the ChCl:o-PDA (1:1) eutectic mixture (1 mL) under magnetic stirring. The resulting mixture was stirred at 80 °C for 8–10 min. The reaction was monitored by TLC and GC/MS analysis. After this time, 2 mL of H_2O were added. The resulting aqueous suspension was then extracted with AcOEt (3 x 2 mL). The organic phases were dried over Na_2SO_4, followed by evaporation under reduced pressure to give the corresponding products 1a–8a. Spectral data were in accordance with the literature [21].

The reaction of benzaldehyde in the ChCl:o-PDA DES to give 1a was scaled up using 20 mol (entry 1, Table 3, footnote c). The reaction was complete in 30 min with 93% isolated yield after simple water addition (10 mL) and extraction with 10 mL ethyl acetate.

General Procedure for the Synthesis of 1,2-Substituted Benzimidazoles 1b–8b in the DES ChCl:o-PDA (1:1).

The appropriate aldehyde (2 mmol) was added to the ChCl: o-PDA (1:1) eutectic mixture (1 mL) under magnetic stirring. The resulting mixture was stirred at 80 °C for 8–10 min. The reaction was monitored by TLC and GC/MS analysis. After this time, 2 mL of H_2O were added. The resulting aqueous suspension was then extracted with AcOEt (3 x 2 mL). The organic phases were dried over Na_2SO_4, followed by evaporation under reduced pressure to give the corresponding products 1b–8b. Spectral data were in accordance with the literature [21].

MS(EI) Spectra of Benzimidazole Derivatives

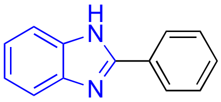

1a

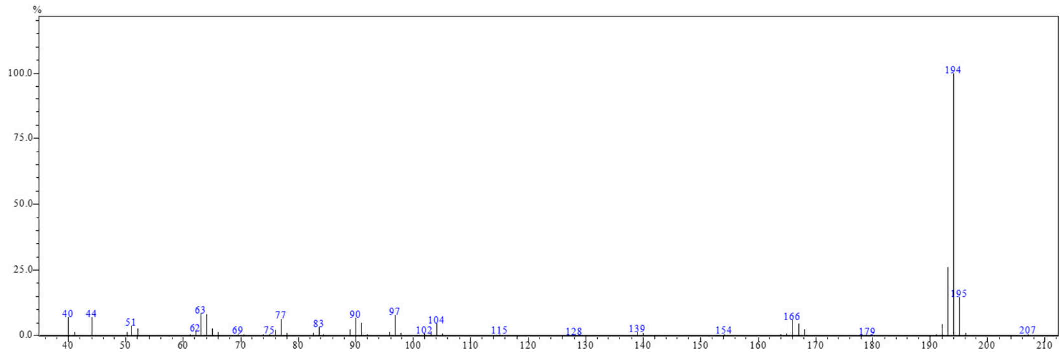

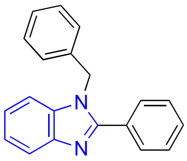

1b

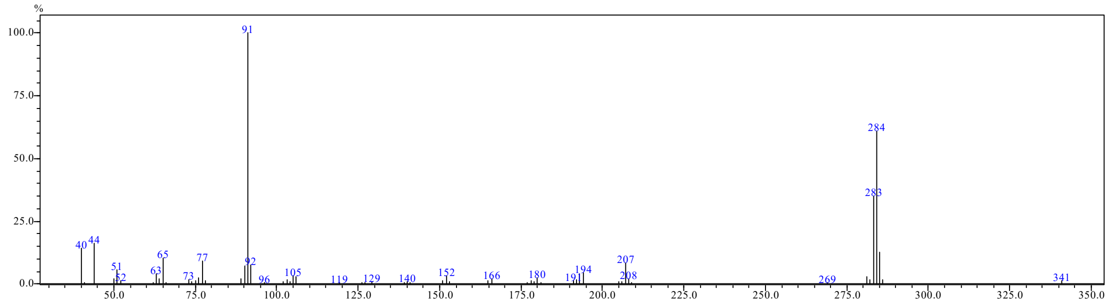

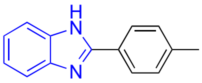
H
N
N

2a

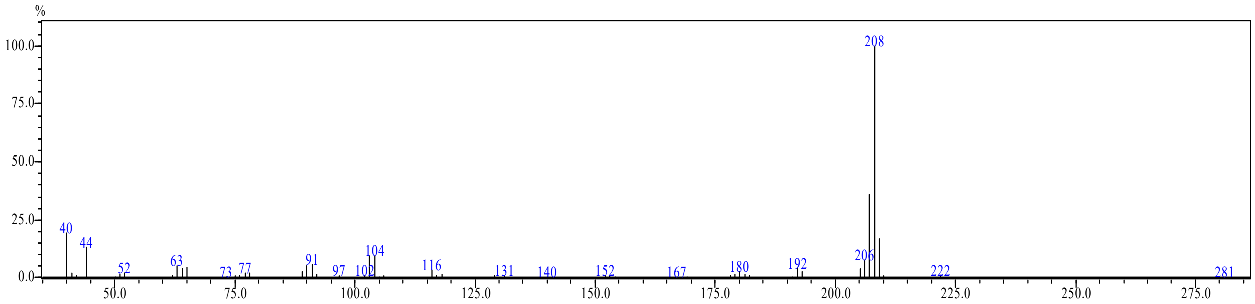
%
100.0
75.0
50.0
25.0
0.0
208
40
44
52
63
73
77
91
97
102
104
116
131
140
152
167
180
192
206
222
281
50.0
75.0
100.0
125.0
150.0
175.0
200.0
225.0
250.0
275.0

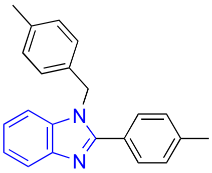
N
N

2b

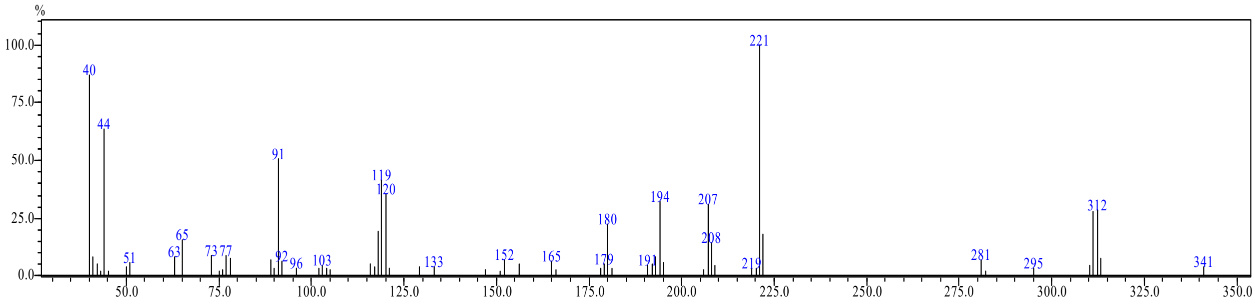
%
100.0
75.0
50.0
25.0
0.0
221
40
44
91
119
120
194
207
180
312
208
51
63
65
73
77
92
96
103
133
152
165
179
191
219
281
295
341
50.0
75.0
100.0
125.0
150.0
175.0
200.0
225.0
250.0
275.0
300.0
325.0
350.0

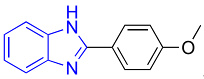

3a

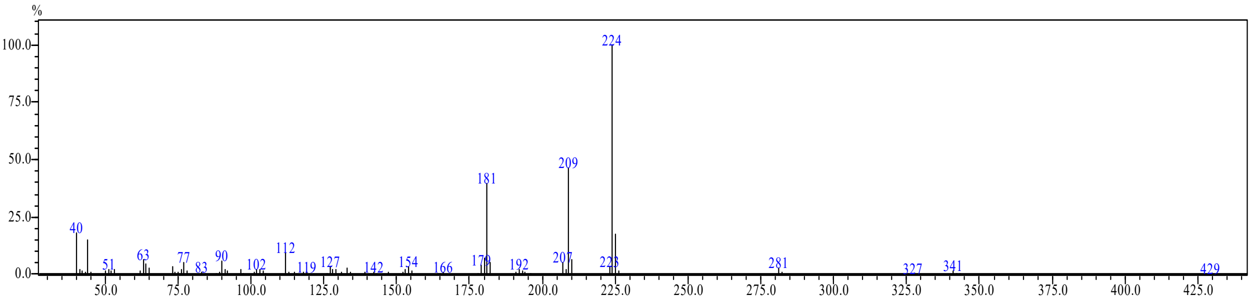
%
100.0
75.0
50.0
25.0
0.0
224
209
181
40
51
63
77
83
90
102
112
119
127
142
154
166
179
192
207
223
281
327
341
429
50.0 75.0 100.0 125.0 150.0 175.0 200.0 225.0 250.0 275.0 300.0 325.0 350.0 375.0 400.0 425.0

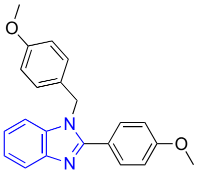

3b

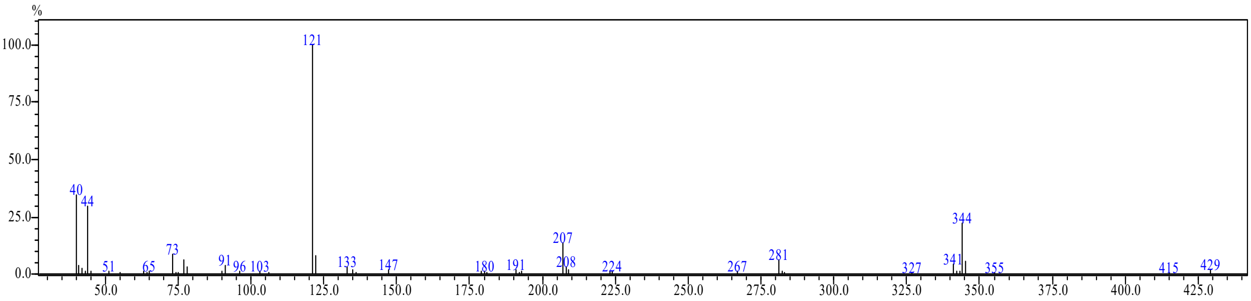
%
100.0
75.0
50.0
25.0
0.0
121
40
44
51
65
73
91
96
103
133
147
180
191
207
208
224
267
281
327
341
344
355
415
429
50.0 75.0 100.0 125.0 150.0 175.0 200.0 225.0 250.0 275.0 300.0 325.0 350.0 375.0 400.0 425.0

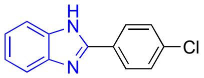
H
N
N
Cl

7a

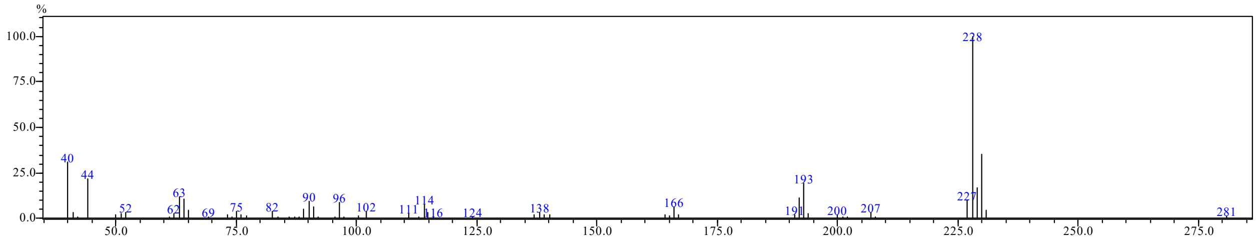
%
100.0
75.0
50.0
25.0
0.0
50.0
75.0
100.0
125.0
150.0
175.0
200.0
225.0
250.0
275.0
228
40
44
52
62
63
69
75
82
90
96
102
111
114
116
124
138
166
193
200
207
227
281

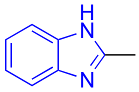

5a

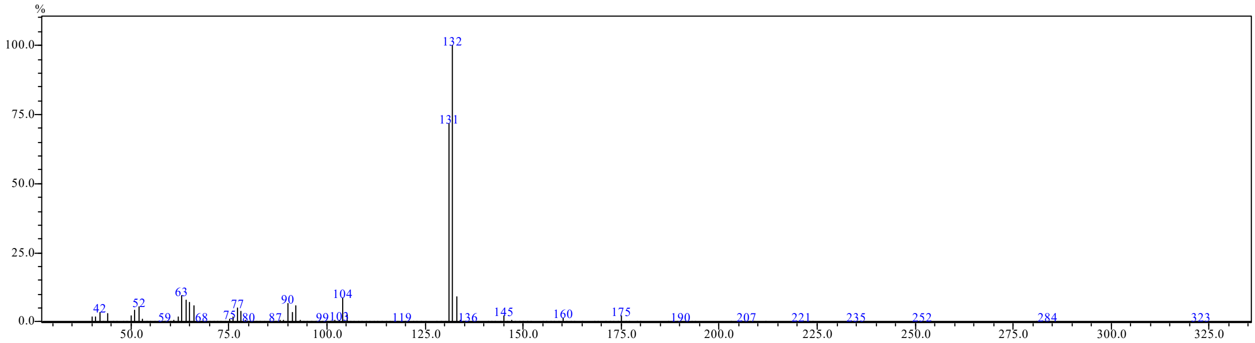

%
100.0
75.0
50.0
25.0
0.0
132
131
42
52
59
63
68
75
77
80
87
90
99
103
104
119
136
145
160
175
190
207
221
235
252
284
323
50.0
75.0
100.0
125.0
150.0
175.0
200.0
225.0
250.0
275.0
300.0
325.0

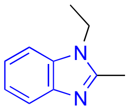

5b

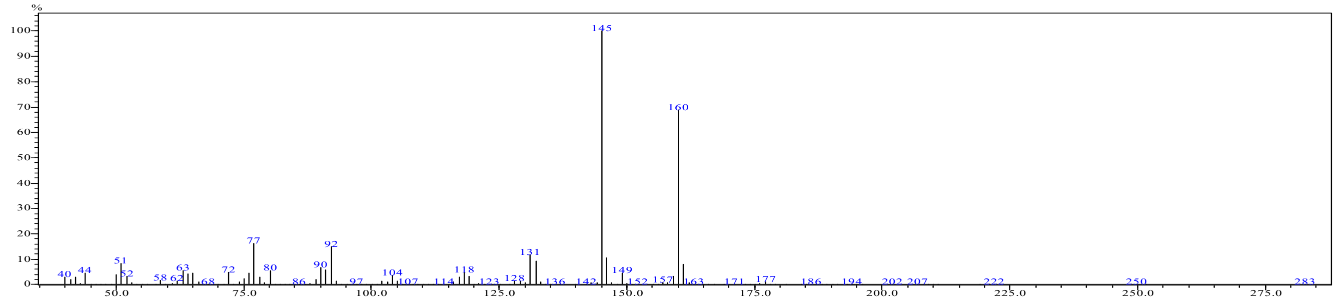

%
100
90
80
70
60
50
40
30
20
10
0
145
160
40
44
51
52
58
63
68
72
77
80
86
90
92
97
104
107
114
118
123
128
131
136
142
149
152
157
163
171
177
186
194
202
207
222
250
283
50.0
75.0
100.0
125.0
150.0
175.0
200.0
225.0
250.0
275.0

Differential Scanning Analysis (DSC) of ChCl:o-PDA (1:1)

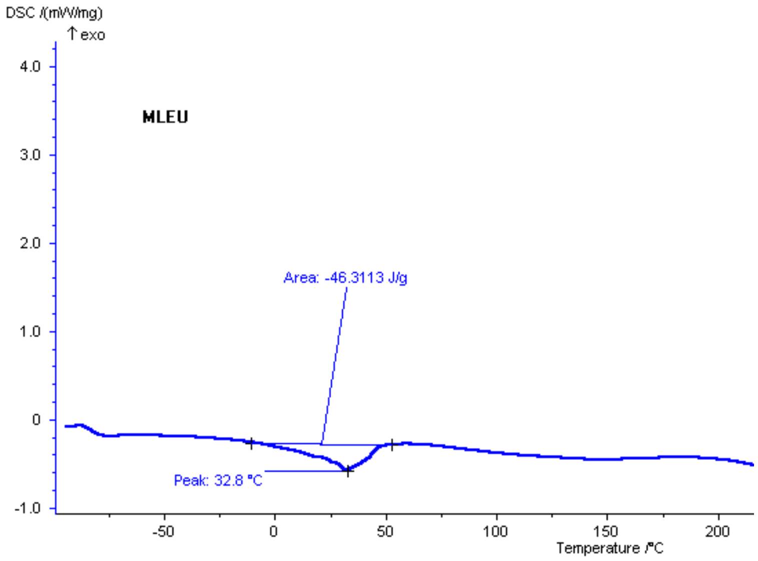

Differential Scanning Analysis (DSC) of Pure o-PDA

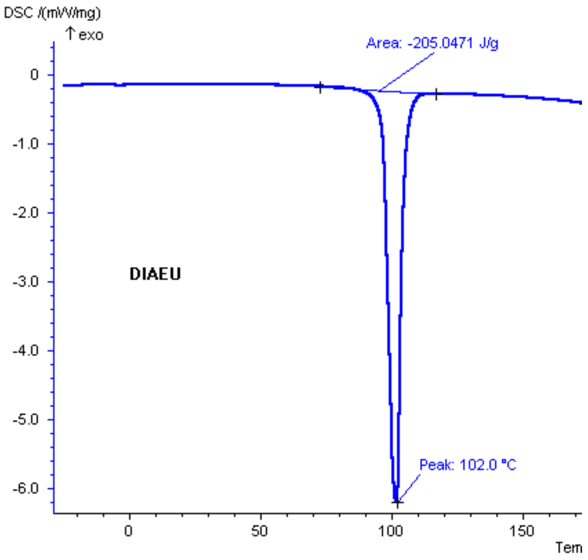
DSC /(mW/mg)
↑ exo
Area: -205.0471 J/g
0
-1.0
-2.0
-3.0
-4.0
-5.0
-6.0
DIAEU
Peak: 102.0 °C
0
50
100
150
Tem
